# Pindborg Tumor—An Uncommon Odontogenic Tumor Detected by 68Ga-DOTATOC

**DOI:** 10.3390/diagnostics12020389

**Published:** 2022-02-02

**Authors:** Anna Herden, Deema Sabtan, Katja Warnecke, Christian Doll, Christian Furth

**Affiliations:** 1 Department of Nuclear Medicine, Chariteé—Universitätsmedizin Berlin, Corporate Member of Freie Universität Berlin, Humboldt-Universität zu Berlin, Augustenburger Platz 1, 13353 Berlin, Germany; anna.herden@charite.de; 2 Institute of Pathology, Chariteé—Universitätsmedizin Berlin, Corporate Member of Freie Universität Berlin, Humboldt-Universität zu Berlin, Chariteéplatz 1, 10117 Berlin, Germany; deema.sabtan@charite.de; 3 Departement of Internal Medicine, Rheumatology, Clinical Immunology and Osteology, Immanuel Krankenhaus Berlin-Wannsee, Koenigstrasse 63, 14109 Berlin, Germany; katja.warnecke@immanuelalbertinen.de; 4 Department of Oral and Maxillofacial Surgery, Chariteé—Universitätsmedizin Berlin, Corporate Member of Freie Universität Berlin, Humboldt-Universität zu Berlin, Augustenburger Platz 1, 13353 Berlin, Germany; christian.doll@charite.de

**Keywords:** Tumor-induced-osteomalacia (TIO), ^68^Ga-DOTATOC, Pindborg tumor

## Abstract

A 62-year-old-woman with a suspected Tumor-induced-osteomalacia (TIO), a rare neoplastic syndrome that results in renal phosphate wasting with hypophosphatemia, underwent ^68^Ga-DOTATOC PET/CT on the suspicion of a mesenchymal tumor producing Fibroblast growth factor 23 (FGF23). Imaging revealed a small osteolytic, somatostatin receptor (SSTR) positive lesion containing calcifications in the alveolar process of the maxilla. No other SSTR-positive focus was found. A biopsy was performed by an oral and maxillofacial surgeon that revealed a calcifying epithelial odontogenic tumor (Pindborg tumor). This case shows that epithelial odontogenic tumors as an uncommon benign tumor entity can also be SSTR-positive.

## Introduction

**Figure 1:**
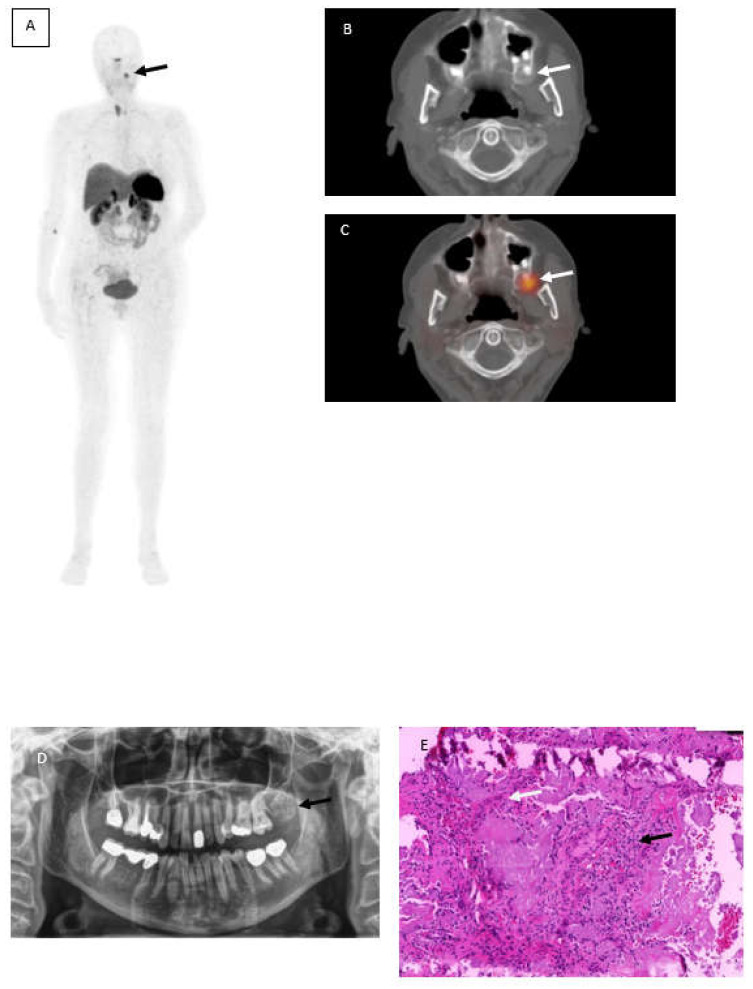
^68^Ga-DotaTOC PET/CT showing focally increased tracer uptake maxillary left (**A**) black arrow corresponding with a well circumscribed radiopaque area (**B**,**C**) white arrows. Orthopantomogramm shows a mixed radiopaque, well-defined mass at corresponding location (**D**) and histopathological photograph (**E**) hematoxylin and eosin-stained sections revealed several panCK positive sheets of eptithelial cells (black arow) interspersed with eosinophilic amyloid-like material (white arrow).

A 62-year-old woman with suspected Tumor-induced-osteomalacia (TIO) because of biochemical evidence of Fibroblast growth factor 23 (FGF23) after several fractures caused by low bone density underwent somatostatin receptor imaging PET/CT (SSTR-PET/CT) using ^68^Ga-DotaTOC. Although rare, in most cases the underlying cause of TIO is a small mesenchymal neoplasm that is difficult to detect without hybrid imaging [1] because of the small size, the different locations and that it generally causes no pain symptoms. Therefore, computed tomography and magnetic resonance imaging are often incapable in detecting those tumor entities. In this case,^68^Ga-DotaTOC PET/CT revealed a focally increased tracer uptake maxillary left (Figure 1A, black arrow) corresponding with a well circumscribed radiopaque area at region 27 to 29 (Figure 1B and C, white arrows). Within this area, a slightly increased radiolucency with parts of scattered areas due to lose of calcification needed to be obtained. No further SSTR-positive foci were found. An orthopantomogram showed a mixed radiopaque, well-defined mass at corresponding location (Figure 1D). A biopsy was performed by an oral and maxillofacial surgeon leading to a partial resection of the tumor. Later on, an entire resection of the tumor was completed by the partial resection of the Maxilla.

Photomicrographs including hematoxylin and eosin-stained sections (Figure 1, Image E) led to the diagnosis of a calcifying epithelial odontogenic tumor (Pindborg tumor). The hematoxylin and eosin staining revealed several panCK positive sheets of epithelial cells (black arrow) interspersed with eosinophilic amyloid-like material (white arrow) surrounded by Vimentin positive desmoplastic stromal reaction.

A Pindborg tumor is an uncommon, benign epithelial odontogenic tumor which was first described by Pindborg in 1955 [2]. It is locally aggressive and accounts for 1% of the total odontogenic tumors with recurrence in 14% of cases [3]. Clinically, it presents as a slow-growing, painless expansile hard bony swelling, largely associated with an impacted tooth [4].

This case shows that epithelial odontogenic tumors as an uncommon benign tumor entity can also be SSTR-positive. To the best of our knowledge, there are no known cases of Pindborg tumors being SSTR positive or detected with other radioligands.

Although we thought that our case was just a rare SSTR-positive pitfall, amazingly, after partial resection, the level of FGF-23 in blood decreased and normalized after total resection. Therefore, it might be possible that besides mesenchymal tumors known for producing FGF-23 [5], epithelial odontogenic tumors might be responsive for high FGF-23 levels in blood. Although the first histopathological results by additional FGF23-mutation analysis of the tumor-DNA showed no relevant mutation, further investigations need to be conducted, since FGF23-mutation is only 1 of several possibilities for an overexpression of FGF23 [6,7,8].

## References

[1] Clifton-Bligh R.J., Hofman M.S., Duncan E., Sim I.-W., Darnell D., Clarkson A., Wong T., Walsh J.P., Gill A.J., Ebeling P.R. (2013). Improving Diagnosis of Tumor-Induced Osteomalacia with Gallium-68 DOTATATA PET/CT. J. Clin. Endocrinol. Metab..

[2] Angadi P.V., Rekha K. (2011). Calcifying Epithelial Odontogenic Tumor (Pindborg Tumor). Head Neck Pathol..

[3] Gotmare S.S., Treville P., Shetty S., Kesarkar K.S. (2018). Pindborg tumor: Pathology with special stains. Indian J. Pathol. Microbiol..

[4] Caliaperoumal S.K., Gowri S., Dinakar J. (2016). Pindborg Tumor. Contemp. Clin. Dent..

[5] Florenzano P., Hartley I.R., Jimenez M., Roszko K., Gafni R.I., Collins M.T. (2021). Tumor-Induced Osteomalacia. Calcif. Tissue Int..

[6] Katoh M. (2016). FGFR inhibitors: Effects on cancer cells, tumor microenvironment and whole-body homeostasis (Review). Int. J. Mol. Med..

[7] Onishi T., Umemura S., Shintani S., Ooshima T. (2008). Phex mutation causes overexpression of FGF23 in teeth. Arch. Oral. Biol..

[8] Carter J.M., Caron B.L., Dogan A., Folpe A.L. (2015). A novel chromogenic in situ hybridization assay for FGF23 mRNA in phosphaturic mesenchymal tumors. Am. J. Surg. Pathol..

